# A broad spectrum screening of Schmallenberg virus antibodies in wildlife animals in Germany

**DOI:** 10.1186/s13567-015-0232-x

**Published:** 2015-09-23

**Authors:** Susan Mouchantat, Kerstin Wernike, Walburga Lutz, Bernd Hoffmann, Rainer G. Ulrich, Konstantin Börner, Ulrich Wittstatt, Martin Beer

**Affiliations:** Junior Research Group Wildlife Diseases, Friedrich-Loeffler-Institut (FLI), Südufer 10, 17493 Greifswald, Insel Riems Germany; Institute of Diagnostic Virology, Friedrich-Loeffler-Institut (FLI), Südufer 10, 17493 Greifswald, Insel Riems Germany; Institute of Wildlife Research, Pützchens Chaussee 228, 53229 Bonn, Germany; Institute for Novel and Emerging Infectious Diseases, Friedrich-Loeffler-Institut (FLI), Südufer 10, 17493 Greifswald, Insel Riems Germany; Leibniz Institute for Zoo and Wildlife Research (IZW), Alfred-Kowalke-Str. 17, 10315 Berlin, Germany; State Laboratory Berlin-Brandenburg (LLBB), Invalidenstr. 60, 10557 Berlin, Germany

## Abstract

To identify native wildlife species possibly susceptible to infection with Schmallenberg virus (SBV), a midge-transmitted orthobunyavirus that predominantly infects domestic ruminants, samples from various free-living ruminants, but also carnivores, small mammals and wild boar were analyzed serologically. Before 2011, no SBV-specific antibodies were detectable in any of the tested species, thereafter, a large proportion of the ruminant population became seropositive, while every sample taken from carnivores or small mammals tested negative. Surprisingly, SBV-specific-antibodies were also present in a large number of blood samples from wild boar during the 2011/2012 and 2012/2013 hunting seasons. Hence, free-ranging artiodactyls may play a role as wildlife host.

## Introduction, methods and results

Schmallenberg virus, a midge-transmitted orthobunyavirus, was initially detected in domestic ruminants near the German/Dutch border in late 2011 [1]. Since then, the virus spread very rapidly among European livestock. After the first vector season a very high seroprevalence of approximately 70% to nearly 100% was observed in domestic ruminants in the centre of the epidemic in North-Western Germany, the Netherlands and Belgium [2-5]. In the following vector season, SBV still circulated in that area, but at a much lower level [6], and in 2013, cases of viral genome detection were reported only sporadically to the German Animal Disease Reporting System (TSN). However, in summer and autumn 2014, SBV reappeared to a greater extent [7] and the reasons for that observation are not completely elucidated until now. One possible explanation could be the existence of transient reservoir hosts for the virus apart from the major target species. Until now, viral genome or specific antibodies were detected predominantly in domestic and wild ruminants, such as cattle, sheep, goats, mouflon, bison, moose, alpacas, buffalos, bison, and deer [8-12]. However, antibodies were also found in a dog in Sweden [13], and type I interferon receptor knock-out mice are susceptible to an experimental SBV-infection [14]. To examine whether free-living carnivores or small mammals, i.e. rodents and shrews, may be infected by SBV, 339 blood samples from a variety of carnivores (red fox - *Vulpes vulpes*, raccoon dog - *Nyctereutes procyonoides*, raccoon - *Procyon lotor*, marten - *Martes* spp.) as well as 195 samples from small mammals (members of the families Muridae, Cricetidae and Soricidae; approved by the competent authority, LANUV NRW, ref. 8.87-51.05.20.09.210) were collected between 2011 and 2012 and tested for the presence of SBV-specific antibodies. Though the detection of specific antibodies does not inevitably reflect a productive infection, the short viraemia of only a few days [1,15] makes the detection of anti-SBV antibodies to a much more promising diagnostic test system than the detection of the virus itself, especially for epidemiological investigations.

Wild boar (*Sus scrofa*), considered as a reservoir for several viruses of livestock and humans, is the second most abundant ungulate in Europe. Based on official hunting statistics Germany is one of the countries with the highest population densities of wild boar in Europe [16]. In previous investigations neutralizing antibodies against Akabane virus, a member of the Simbu sero-group of the genus *Orthobunyavirus*, were detected in warthogs and bush pigs in Africa [17,18] and in pigs in Taiwan [19].

To investigate whether wild boar are susceptible to an SBV-infection and may serve as a reservoir, a total of 2077 blood samples taken *post mortem* in 2006 and between August 2010 and December 2013 was analyzed for the presence of SBV-specific antibodies. 1646 of the 2077 samples were collected in North Rhine-Westphalia, the German federal state where the first case of SBV-infection was detected [1]. In the 2013/2014 hunting seasons, predominantly young animals (<1 year) were sampled. In addition, samples from European mouflon (*Ovis orientalis musimon*), as a wild sheep the only free-living wild form of susceptible domestic animals in Germany, and further free-living ruminants such as roe deer (*Capreolus capreolus*), fallow deer (*Dama dama*), red deer (*Cervus elaphus*), and sika deer (*Cervus nippon*) were analyzed (Table 1). Blood samples from deer and mouflon as well as wild boar and carnivores were collected in cooperation with local hunters according to the appropriate German legislation. No ethical/welfare authority approval was required as samples were collected post-mortem by the hunters. All blood samples were examined with an indirect or a competitive commercially available SBV-antibody ELISA (ID Screen® Schmallenberg virus Indirect or ID Screen® Schmallenberg virus Competition, both IDvet, Grabels, France) according to the manufacturer’s recommendations. In the indirect ELISA kit an Anti-multi-species IgG-HRP conjugate is included. Samples with a doubtful ELISA result as well as a representative number of samples from each species with positive and negative ELISA results were retested by a standard micro-neutralization assay as described previously [15].

**Table 1:** **Serological results of German wildlife screening for Schmallenberg virus infection**

**Species**	**Hunting season or time period**	**Samples**	**Positive (%)**	**Negative (%)**
Mouflon	2011/2012	4	4 (100)	0
	2012/2013	31	26 (83.87)	5 (16.13)
	2013/2014	9	3 (33.33)	6 (66.67)
Deer^a^	2000/2001	134	0	134 (100)
	2011/2012	136	41 (30.15)	95 (69.85)
	2012/2013	760	278 (36.58)	482 (63.42)
	2013/2014	324	65 (20.06)	259 (79.94)
	2014/2015	4	2 (50)	2 (50)
Carnivores^b^	2011/2012	281	0	281 (100)
	2012/2013	58	0	58 (100)
Small mammals^c^	2011-2012	195	0	195 (100)

The results are divided by species and hunting seasons (huntable animals) resp. time period (small mammals). A hunting season takes from 1^st^ April to 31^st^ March next year.

^a^Roe deer, red deer, sika deer, and fallow deer.

^b^Red fox, marten, badger, raccoon dog, and raccoon.

^c^Rodents, and shrews.

No antibodies against SBV could be detected in samples of the 339 wild carnivores collected from 2011 to 2013, and in samples of the 195 small mammals collected in 2011 and 2012.

In contrast, within this time frame (2011–2012) about 30% of the deer and all 4 tested mouflons were SBV-antibody-positive (Table 1). Furthermore, in the hunting season 2012/2013 antibodies against SBV were detectable in approximately 84% of the mouflons and 37% of the samples from deer. In the following season the seroprevalence declined to about 33% and 20%, respectively (Table 1).

In addition to the samples taken after the presumed date of SBV-introduction into Europe, historical samples collected from wild ruminants (roe deer, red deer, and fallow deer) in Germany before 2011 were analyzed. All 134 samples tested negative in an SBV-specific antibody-ELISA (Table 1). The same holds true for wild boar, every sample taken before autumn 2011 tested negative.

**Figure 1:**
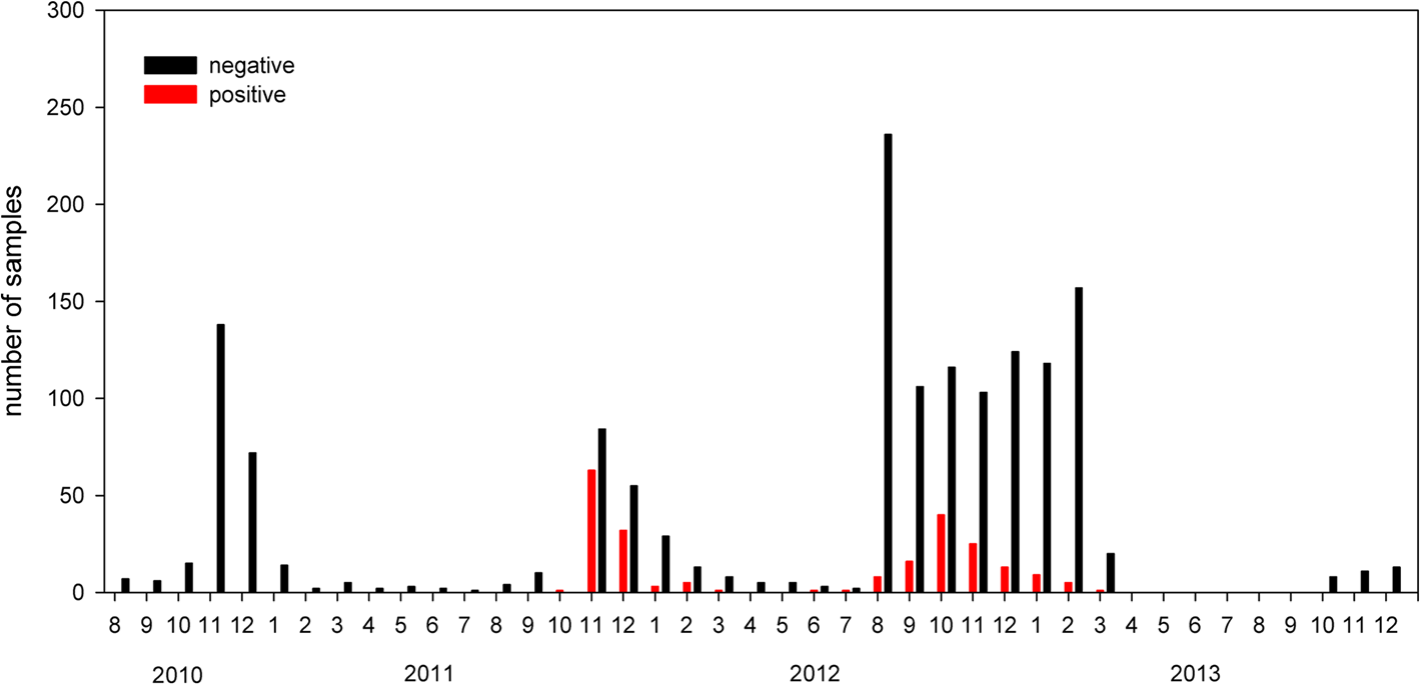
**Frequency of Schmallenberg virus-specific antibodies in wild boar.** Samples were collected between August 2010 and December 2013 and analyzed by a commercially available ELISA resp. serum neutralization test. The number of negative results per month is shown as a black bar and the number of positive results is displayed as a red bar. Only samples with information about months of culling were depicted.

However, from October 2011 onwards, SBV-specific antibodies were frequently also detected in wild boar (Figure 1). In the hunting season 2011/2012, 105 out of 316 samples tested positive (33%), in the following season SBV-specific antibodies were detectable in 11% of the samples (119 out of 1114), while in 2013/2014 all of the analyzed 32 samples scored negative (Figure 1).

A number of samples with positive ELISA results from each species were also confirmed by a highly specific serum neutralization test, and the resulting titers ranged from 1:5 up to 1:30 (mouflon), 1:640 (roe deer), 1:15 (fallow deer), 1:60 (red deer), 1:60 (sika deer), or 1:80 (wild boar).

Nevertheless, during the entire period from 2011 to the 2014–2015 hunting season, malformations were not reported by hunters, neither in wild ruminants, nor in wild boar.

## Discussion

As shown for alpine ungulates or deer hunted in countries that border Germany, SBV is capable to infect several ruminant species [9,11,12]. In the present study, however, SBV-specific antibodies were not only detected in a wide range of ruminants, but also in wild boar which belong to the closely related Suidae family within the order Artiodactyla.

After experimental SBV-infection of domestic pigs, in only a small proportion of animals a temporary seroconversion was observed; neutralization titers that barely reached the limit of detection were measured in a few animals for a short time, while the SBV-specific ELISA scored negative in every case [20]. As opposed to experimentally inoculated domestic pigs, neutralizing anti-SBV antibodies could not be detected in field-collected sera [20]. On the contrary, a large proportion of wild boar tested positive by ELISA in our study, and neutralization titers even exceeded those measured in wild ruminants, i.e. mouflon, fallow deer, red deer, or sika deer. Therefore, and because of the positive results in two independent test systems, and the absence of measurable SBV-specific antibodies before 2011, the year of presumed virus introduction into Europe, unspecific reactions are very unlikely. Especially as the insect vectors responsible for SBV-transmission, such as *Culicoides* midges of the Obsoletus group [21], evidently also feed on members of the Suidae family [22]. The reasons for the obvious differences in the susceptibility of domestic pigs and wild boar to an SBV-infection, however, need to be evaluated in future studies. In this context the possibility has to be considered that midges might feed repeatedly on an individual animal which could induce a measurable immune response also in pigs resp. wild boar. Furthermore, it might be possible that the pathogen is only mechanically transmitted by the vector [18].

Though the applied ELISA tests might cross-react with antibodies against viruses closely related to SBV and the serum neutralization test is in general considered as the most sensitive and specific system for the detection of SBV-specific antibodies [23], only a subset of samples could be tested in this assay. Since the samples were taken from hunted animals under non-sterile conditions in the present study, the quality (bacterial contamination, cytotoxicity) hampered the cell culture-based neutralization assay. However, a good correlation between ELISA results and neutralization titers was observed in every tested sample (data not shown), and the commercially available SBV-ELISAs have been previously successfully applied not only for sera from cattle, sheep or goats, for which they have been originally produced, but also for further species such as wild ruminants, domestic pigs or mice [20,24,25]. Here, the applicability of this test system was demonstrated for wild boar as well.

In keeping with domestic ruminants, SBV was not present in German wildlife until late 2011. Thereafter, a large proportion of seropositive animals was found. The lower seroprevalence in the wild boar population after the 2012/2013 hunting season corresponds to that observed in domestic ruminants such as cattle [6] and further free-living ruminants (Table 1). Most likely caused by a high seroprevalence in the population of susceptible animals after the first vector season, the virus circulated only on a limited scale in the following years resulting in a missing infection of the SBV-naïve young stock which in turn has led to a decline in herd seroprevalence. Apart from the supply with seronegative offspring over the time, a gradual reduction of SBV-specific antibodies in individual animals could be an explanation for the declining herd seroprevalence. However, in other animal species, such as cattle, the titers of anti-SBV antibodies are mostly stable for at least two years [26], and in the present study, predominantly young animals were tested after the 2011/2012 hunting season. SBV-specific antibodies were detectable in a number of those animals in the last years which is not only explainable by maternal antibodies (e.g. for animals older than 6 months), but also by new infections caused by a low level of virus circulation. The annual testing of subadults could show whether this low level of infections will persist in the next years and, if so, it will lead to a renewed virus circulation on a larger scale which might be expected as soon as the level of the specific immunity within the complete population will further decline.

In domestic ruminants, the most important effect of SBV is stillbirth, premature birth and the induction of severe congenital malformations when dams are infected during a critical period of pregnancy [4,27]. Despite the high rate of SBV-infections in the first two years (autumn 2011 and 2012), no aborted, stillborn and/or malformed fawns or boar piglets were reported, neither from German hunters or forest rangers, nor from further European countries such as Belgium or the UK [12,28]. This may be due to the fact, that embryos can only be infected in a critical time of pregnancy i.e. after establishment of the first placentome and before the fetus is immunologically competent. Until now, only one case of a cervine fetus with SBV-typical malformation was found in utero; however, further abnormalities were also visible and an SBV-specific RT-PCR tested negative [29]. Consequently, it remains unclear whether SBV may cause transplacental infection in wild animals with the effects seen in cattle or sheep. This question is difficult to be answered for wildlife because aborted fetuses or unviable newborn malformed animals might be quickly eaten by scavengers, and therefore are extremely difficult to collect. All carnivores, which might be in contact to the virus by eating aborted fetuses and stillborn newborns, tested in contrast to ruminants or wild boar negative for SBV-specific antibodies. In addition to carnivores, free-living shrews and rodents are most likely also not a reservoir for SBV; specific antibodies were not detected in the tested species which display an intact interferon system in contrast to the SBV susceptible type I interferon receptor deficient mice [14,24].

In conclusion, SBV-specific antibodies were detectable in all free-ranging cervids and wild sheep (mouflons) present in Germany, but also in wild boar, indicating that not only ruminants but further members of the artiodactyls are susceptible to an SBV-infection. Hence, free-ranging artiodactyls but not small mammals or wild carnivores may play a role as an additional host in the epidemiology of SBV.
